# Supplementary material for: Effects of Pesticide Intake on Gut Microbiota and Metabolites in Healthy Adults
Source: Int J Environ Res Public Health. 2022 Dec 23;20(1):213. doi: 10.3390/ijerph20010213 (PMC9819155; doi:10.3390/ijerph20010213)
Supplement: Supplementary file 1 [file ijerph-20-00213-s001.zip › ijerph-2123225-supplementary.pdf]

Supplementary information

International Journal of Environmental Research and Public Health
Manuscript type: Article

Title: Effects of pesticide intake on gut microbiota and metabolites in healthy adults

Authors: Jun Ueyama[1,*], Mai Hayashi[1], Masaaki Hirayama[1], Hiroshi Nishiwaki[2], Mikako Ito[2], Isao Saito[1], Yoshio Tsuboi[3], Tomohiko Isobe[4], Kinji Ohno[2]

[1]Department of Pathophysiological Laboratory Sciences, Field of Radiological and Medical Laboratory Sciences, Nagoya University Graduate School of Medicine, 1-1-20 Daiko-minami, Higashi-ku, Nagoya Japan

[2]Division of Neurogenetics, Center for Neurological Diseases and Cancer, Nagoya University Graduate School of Medicine, 65 Tsurumai-cho, Showa-ku, Nagoya, Japan

[3]Department of Neurology, Fukuoka University, 7-45-1 Nanakuma, Jonan-ku, Fukuoka, Japan.

[4]Health and Environmental Risk Division, National Institute for Environmental Studies, 16-2 Onogawa, Tsukuba, Japan

***Corresponding author:** Jun Ueyama (e-mail: ueyama@met.nagoya-u.ac.jp)

Table S1. Food and drinking frequencies, smoking, and supplement intake habits of the participants.

	Never	1–2 times/week	3–5 times/week	Every day	No data
Food and drinking Frequency					
Rice	0	1	1	27	9
Bread	4	5	5	15	9
Pasta	1	20	6	1	10
Potato	1	15	11	2	9
Fish	0	9	15	5	9
Meat	0	7	15	7	9
Milk	9	2	2	15	10
Fermented milk	9	2	3	14	10
Beans	0	6	16	7	9
Fermented bean	9	12	2	6	9
Root vegetables	0	5	10	13	10
Vegetables (others)	0	3	7	19	9
Konjac	1	24	3	1	9
Mushroom	1	8	16	4	9
Seaweed	0	12	12	5	9
Coffee	2	3	4	20	9
Alcohol (except for beer)	24	1	1	2	10
Smoking	Never	19			10
	Past	7			
	Current	2			
Beer (/day)	Mean (range)	0.125 (0–2)			8
Supplement	No	21			8
	Yes	9			

Table S2. Differences in the fecal bacterial component between low- and high-DAP groups (cutoff value: median) using ANCOM and Wilcoxon rank-sum tests.

Genus	ANCOM			Wilcoxon rank-sum test	
	W-statistic	detected 0.8	detected 0.7	*p* value	FDR
Peptococcus	0	FALSE	FALSE	0.009[a]	0.89
Cloacibacillus	0	FALSE	FALSE	0.019[a]	0.89
Agathobacter	111	FALSE	TRUE	0.012[b]	0.89
Lachnospiraceae.ND3007.group	0	FALSE	FALSE	0.028[b]	0.89
Coprococcus	0	FALSE	FALSE	0.041[b]	0.89

[a]Lower relative abundance in the high-OP exposure group. [b]Higher relative abundance in the high-OP exposure group. FDR, false discovery rate.

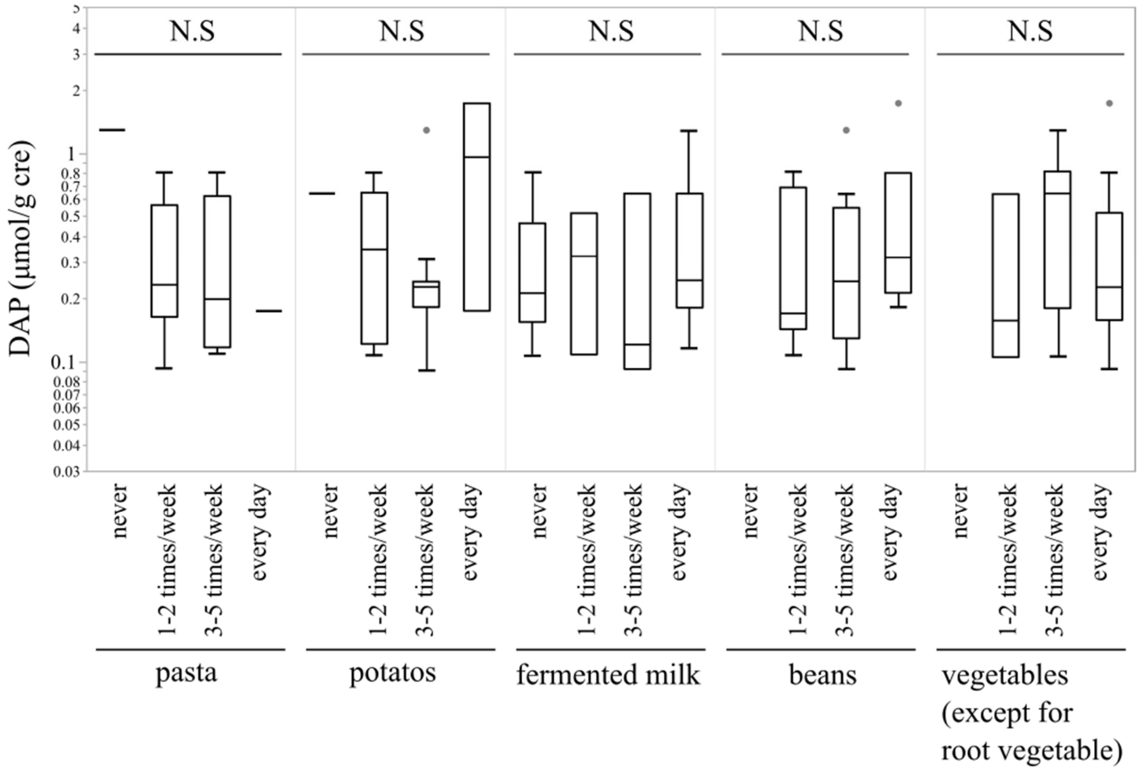

Figure S1. Outlier box plots of total concentrations of urinary DAP (µmol/g cre) for each food intake frequency related to fecal acetate concentration in stepwise regression analysis (model 2). Whiskers are drawn to the furthest point within 1.5 × the inter quartile range from the box. N.S, no significant differences (Kruskal-Wallis test).
